# Respiratory sleep disorders, nasal obstruction and enuresis in children with non-syndromic Pierre Robin sequence

**DOI:** 10.1016/j.bjorl.2021.05.002

**Published:** 2021-05-28

**Authors:** Fábio Luiz Banhara, Inge Elly Kiemle Trindade, Ivy Kiemle Trindade-Suedam, Marilyse de Bragança Lopes Fernandes, Sergio Henrique Kiemle Trindade

**Affiliations:** a Universidade de São Paulo (USP), Hospital de Reabilitação de Anomalias Craniofaciais (HRAC), Unidade de Estudos do Sono do Laboratório de Fisiologia, Bauru, SP, Brazil; b Universidade de São Paulo (USP), Faculdade de Odontologia de Bauru, Bauru, SP, Brazil; c Universidade de São Paulo (USP), Hospital de Reabilitação de Anomalias Craniofaciais (HRAC), Seção de Otorrinolaringologia, Bauru, SP, Brazil; d Universidade Nove de Julho, Curso de Medicina, Bauru, SP, Brazil

**Keywords:** Obstructive sleep apnea, Nasal obstruction, Enuresis, Pierre Robin syndrome

## Abstract

**Introduction:**

Obstructive sleep apnea is highly prevalent in non-syndromic Pierre Robin sequence patients. Studies have found a probable relationship between obstructive sleep apnea and nasal obstruction and between obstructive sleep apnea and enuresis. Assessment of the relationship between these variables in non-syndromic Pierre Robin sequence patients is scarce.

**Objective:**

The present study aims to evaluate the relationship between symptoms of obstructive sleep apnea, nasal obstruction and enuresis, determining the prevalence of symptoms suggestive of these conditions, in schoolchildren with non-syndromic Pierre Robin sequence, and describe the prevalence of excessive daytime sleepiness habitual snoring and voiding dysfunction symptoms associated with enuresis.

**Methods:**

This was a prospective analytical cross-sectional study developed at a reference center. Anthropometric measurements and a structured clinical interview were carried out in a sample of 48 patients. The instruments “sleep disorders scale in children” “nasal congestion index questionnaire” (CQ-5), and the “voiding dysfunction symptom score questionnaire” were used. Statistical analysis was performed for *p* < 0.05.

**Results:**

Positive “sleep disorders scale in children” scores for obstructive sleep apnea and CQ-5 for nasal obstruction were observed in 38.78% and 16.33%, respectively. Enuresis was reported in 16.33% of children, being characterized as primary in 71.43% and polysymptomatic in 55.55%; according to the “voiding dysfunction symptom score questionnaire”. There was a significant relationship between nasal obstruction and obstructive sleep apnea symptoms (*p* < 0.05), but no significance was found between obstructive sleep apnea symptoms and enuresis, and between nasal obstruction and enuresis. The prevalence of excessive daytime sleepiness was 12.24% and of habitual snoring, 48.98%. A family history of enuresis, younger age in years and a positive “voiding dysfunction symptom score questionnaire” score were associated with a higher prevalence of enuresis (*p* < 0.05).

**Conclusion:**

Children with non-syndromic Pierre Robin sequence are at high risk for obstructive sleep apnea symptoms and habitual snoring, with a correlation being observed between nasal obstruction and obstructive sleep apnea symptoms. In addition, the study showed that non-syndromic Pierre Robin sequence, obstructive sleep apnea and nasal obstruction symptoms were not risk factors for enuresis in these patients.

## Highlights

• There was a correlation between the presence of nasal obstruction and obstructive sleep apnea symptoms.
• The prevalence of excessive daytime sleepiness, nasal obstruction and enuresis did not differ from those found in the general pediatric population.
• A family history of enuresis, younger age and a positive score on the “dysfunctional voiding scoring system” were associated with a higher prevalence of enuresis.
• The presence of non-syndromic Pierre Robin sequence, obstructive sleep apnea symptoms and nasal obstruction were not risk factors for the occurrence of enuresis in the studied population.

## Introduction

Pierre Robin sequence (PRS) is a congenital craniofacial anomaly characterized by micro/retrognathia, glossoptosis, and, in most cases, is associated with cleft palate. With a prevalence rate ranging from 1:5600 to 1:30,000 births, PRS determines severe eating difficulties and respiratory complications, showing in up to 60% of cases, an association with syndromic conditions, increasing the complexity of its clinical management.^1^, ^2^

There is a high propensity for children with PRS to develop respiratory sleep disorders (RSD) due to the reduction of the pharyngeal area determined by mandibular retraction and retroposition of the tongue.^1^, ^2^, ^3^ Neonates with PRS have a high prevalence of obstructive sleep apnea (OSA), affecting 85% of them in the first year of life.^3^ In individuals with PRS in the age group between 1 and 18 years, OSA prevalence is around 22%, indicating a gradual improvement over the years, proportional to the increase in pharyngeal dimensions.^1^ However, it is noteworthy that the prevalence is above the average identified in the overall pediatric population, which varies between 5% and 13%.^4^, ^5^

OSA characteristics include frequent episodes of partial or total obstruction of the upper airways (UA) during sleep, followed by hypoxemia and hypercapnia, as well as frequent awakenings that interfere with the quality of sleep.^6^ Recurrent respiratory obstructions can be characterized as obstructive sleep apnea syndrome (OSAS) when associated with clinical signs and symptoms such as severe snoring and excessive daytime sleepiness (EDS).^7^ Severe OSAS is clinically important because it is associated with a higher risk for cardiovascular complications and overall mortality in adulthood. In childhood, it is related to concentration difficulties, irritability and reduced school performance.^6^, ^7^, ^8^

Several studies have indicated an association between nasal obstruction and increased risk of RSD, with a prevalence of this symptom being observed in up to 50% of non-syndromic patients diagnosed with OSA.^9^, ^10^, ^11^, ^12^ It is speculated that the airflow decreases during sleep, due to nasal pathologies, generates progressive negative pressures in the upper airways (UA), facilitating pharyngeal collapse, and, consequently, snoring, obstructive apnea, sleep fragmentation and excessive daytime sleepiness.^10^ The relationship between the nasal obstruction intensity and greater severity and occurrence of OSA, however, remains controversial.^13^, ^14^ Patients with cleft palate have smaller internal nasal and pharyngeal dimensions, constituting a special group, when associated with PRS, since they have significant retrognathia. Thus, patients with PRS show morphological alterations that increase the risk of OSA at different stages of life.^11^, ^15^ Moreover, it should be noted that the primary corrective surgery of the palate may further compromise the nasal and pharyngeal dimensions, generating a higher risk for RSD in this population.^6^, ^11^, ^16^, ^17^

Although controversial, recent evidence suggests a causal relationship between OSA and enuresis.^18^, ^19^ Enuresis is defined as the involuntary and intermittent loss of urine in one or more episodes during sleep, being considered pathological in children over 5 years of age, when it occurs at least twice, in a period of up to 3 months.^11^ Enuresis has psychosocial implications for children and families, leading to impaired self-esteem and poor sleep quality.^18^, ^19^, ^20^

A previous study showed that individuals with cleft lip and palate have a three-fold higher chance of enuresis when compared to the overall pediatric population.^11^

To date, few studies have aimed to identify the prevalence and the relationship between symptoms of obstructive sleep apnea, nasal obstruction and enuresis in the population of school-age children with non-syndromic Pierre Robin sequence (PRS); thus, the present study aims to evaluate the relationship between symptoms of OSA, nasal obstruction and nocturnal enuresis, determining the prevalence of these conditions, in patients between 6 and 12 years of age with non-syndromic PRS; and to describe and analyze the symptoms of excessive daytime sleepiness, habitual snoring and the voiding symptoms associated with enuresis.

## Methods

This is a prospective cross-sectional analytical study carried out in a reference center, after approval by the Ethics Committee for Research in Human Subjects of the aforementioned center (Opinion n. 3.417.073, CAE: 15544019.8.0000.544).

The participants were recruited by convenience sampling, upon their routine consultations, resulting in 48 children previously submitted to primary corrective surgeries, aged 6–12 years, with non-syndromic PRS.

Sample calculation was performed considering a prevalence of RSD of 34%,^11^ obtained through questionnaires applied to the population with non-syndromic cleft lip and palate (NSCLP) and in a pediatric population with non-syndromic PRS of 43%.^21^ As the total population of children with PRS that regularly attended the institution when this study was carried out comprised 250 children, the sample size resulted in 36 participants, considering an expected prevalence of 43% of OSA, with a margin of error of 15% and test power of 80%.

The inclusion criteria were: patients with PRS not associated with syndromes or anomalies, aged between 6 and 12 years and adherence to the research by signing the Free and Informed Consent Term by the parents and the Free and Informed Assent Term by the children. Children with associated genetic syndromes, undergone osteogenic distraction of the mandible or tracheostomy, those with disorders or other malformations (neurological, cardiac, anorectal, urological or spinal cord disorders), under chronic use of medications or recent use of antibiotic therapy for upper respiratory tract infections, those who had a urinary tract infection less than 12 months before and with evidence of urinary dysfunction of neurological cause were excluded from the sample.

Data collection was carried out through a structured clinical interview in a single moment, always by the main researcher, in a private environment, with the research participant and their caregiver, during routine outpatient clinical treatment, according to the therapeutic plan implemented by the institution professionals. Initially, data were collected regarding the sociodemographic profile, clinical and surgical history, and anthropometric measurements were performed, including weight, height and BMI Z-score.^22^ The data were evaluated according to the values ​​proposed by the WHO using the WHO AnthroPlus software^23^ and the participants were classified according to their nutritional status.^24^ Additionally, the ratio between waist circumference and height (WC/H) was evaluated as a complementary criterion for obesity and visceral obesity assessment in the population. A high risk for visceral obesity was considered when the WC/H ratio was ≥0.5 and a low risk was considered when the WC/H ratio was <0.5.^25^

The instruments used to assess OSA, nasal obstruction and enuresis symptoms were: SDSC questionnaire-subscale RSD, translated and validated into Portuguese for the investigation of OSA symptoms and habitual snoring, with scores ≥7 being positive for OSA and, positive for habitual snoring, the answer to item 3 of the referred subscale, with an index ≥3 times a week.^26^, ^27^ For the assessment of excessive daytime sleepiness, the SDSC questionnaire-subscale EDS, validated into Portuguese was used, with scores ≥19 being considered positive.^26^, ^27^ For the evaluation of nasal obstruction, the CQ5 questionnaire was used, adapted and validated into Portuguese, with an extended evaluation in 30 days, with scores ≥6 being considered positive.^11^, ^28^, ^29^ The investigation of enuresis and lower urinary tract dysfunction was performed using the Voiding Dysfunction Symptom Score (VDSS) questionnaire, adapted and validated into Portuguese, including three specific questions about the presence and frequency of enuresis episodes, according to a study by Fernandes et al.^11^, ^30^, ^31^, ^32^ The presence of one or more episodes over 3 months and at least one episode in the last 30 days were considered positive for enuresis.

### Statistical analysis

Quantitative variables were described by position and dispersion measures, such as mean, median, minimum, maximum, interquartile range and standard deviation. Qualitative variables were described as percentage and absolute frequencies. The continuous quantitative variables of two subgroups were compared using the Mann–Whitney tests, according to the normality of the variables. The Chi-Square test was used to determine the dispersion value in the nominal categorical variables and to evaluate their association. Qualitative variables were compared using Fisher's exact test. All analyses were performed with a significance level of 5%.

The prevalence between studies was compared using the two-proportion comparison test, and the prevalence ratio (PR) was calculated, which indicates how much the prevalence in one study is higher than that of another one (the farther from 1, the greater the difference between studies). For values ​​of *p* > 0.05, a prevalence ratio of 1 was inferred.

## Results

Initially, 65 children were selected to participate in the study, according to their scheduled return appointment during the proposed period of data collection, and non-syndromic PRS in the age group of 6–12 years, through previous screening of medical records. Of these, 6 children were excluded from the study due to clinical criteria, 4 refused to participate in the study and 7 missed the scheduled appointments, resulting in a sample of 48 children.

The 48 children with non-syndromic PRS who comprised the sample had a mean age of 9.15 (SD ± 2.44) years, with self-declared white skin color/ethnicity (n = 42, 87.50%), of which 50% were females (n = 24). Only two children (4.17%) had no cleft palate associated with PRS.

Regarding the surgical history, the mean primary palatoplasty in months was 14.28 (±3.88) and secondary palatoplasty in months was 67.58 (±43.42). The most frequently used technique in the primary palatoplasty was Von Langenbeck technique (36.96% of the cases), followed by Furlow technique (34.78%). In the secondary palatoplasty, to which 12, 25% of the sample was submitted, the most frequently used was Von Langenbeck technique (41.67%). Regarding adenoidectomy and/or tonsillectomy, the majority (85.42%) of the assessed children had not had these procedures, and 47.92% underwent maxillary expansion procedures aiming to adapt the upper dental arch for orthodontic treatment.

As for the anthropometric and nutritional classification, the mean BMI Z-score was −0.03 (±1.20), with a predominant normal weight nutritional profile in 75% of the children. Additionally, 85.42% of the patients had a WC/H ratio <0.5, demonstrating low rates of central obesity.

The analysis of the differences between the groups with symptoms of OSA, nasal obstruction and enuresis related to the variables gender, secondary palatoplasty, previous adenotonsillectomy, BMI Z-score and WC/H ratio did not show statistical significance (*p* > 0.05) as observed in Table 1. This fact confirms the sample homogeneity and indicates the absence of influence of surgical and anthropometric characteristics on the assessed results.

**Table 1:** Prevalence of symptoms of obstructive sleep apnea (OSA), nasal obstruction (NO) and enuresis in the samples of children with non-syndromic Pierre Robin sequence, and variations regarding gender, age, secondary palatoplasty, adenoidectomy and/or tonsillectomy surgery, WC/H ratio and BMI-Z score.

Variables	Prevalence								
	OSA, n (%)			NO, n (%)			Enuresis, n (%)		
	Yes	No	*p*	Yes	No	*p*	Yes	No	*p*
	n = 19 (38.78)	n = 29 (61.22)		n = 8 (16.33)	n = 40 (83.67)		n = 8 (16.33)	n = 40 (83.67)	
Gender									
Male	12 (63.15)	12 (41.38)	0.238^a^	3 (37.50)	21 (52.50)	0.701^b^	5 (62.50)	19 (47.50)	0.701^b^
Female	7 (36.85)	17 (58.62)		5 (62.50)	19 (47.50)		3 (37.50)	21 (52.50)	
Age (years)^c^									
Mean ± SD	9.26 ± 2.62	9.07 ± 2.36	0.705	11.13 ± 2.10	8.75 ± 2.33	0.009	7.50 ± 2.00	9.48 ± 2.41	0.035
Median (25%–75% quartiles)	9.00 (6.25–12.00)	9.00 (6.00–11.25)		12.00 (11.50–12.00)	6.00 (6.00–11.00)		6.50 (6.00–9.00)	9.50 (7.00–12.00)	
Secondary palatoplasty^b^									
Yes	4 (21.06)	8 (27.59)	1.000	2 (25.00)	10 (30.00)	1.000	3 (37.50)	9 (22.50)	1.000
No	15 (78.94)	21 (72.41)		6 (75.00)	28 (70.00)		5 (62.50)	31 (77.50)	
Previous A and/or T b									
Yes	2 (10.53)	5 (17.25)	0.687	1 (12.50)	6 (15.00)	1.000	3 (37.50)	4 (10.00)	0.080
No	17 (89.47)	24 (82.75)		7 (87.50)	34 (85.00)		5 (62.50)	36 (90.00)	
WC/H ratio^b^									
<0.5	17 (89.47)	24 (82.75)	0.687	7 (87.50)	34 (85.00)	1.000	7 (87.50)	34 (85.00)	1.000
≥0.5	2 (10.53)	5 (17.25)		1 (12.50)	6 (15.00)		1 (12.50)	6 (15.00)	
BMI Z-score d									
Mean ± SD	−0.01 (±1.03)	−0.04 ± 1.32	0.940	0.17 (±0.87)	−0.07 ± 1.26	0.608	0.59 (±0.89)	−0.16 ± 1.23	0.108
Median (25%–75% quartiles)	0.00 (−0.83 to 0.73)	−0.09 (−0.71 to 0.71)		0.62 (−0.50 to 0.75)	−0.09 (−0.71 to 0.65)		0.69 (0.05–1.02)	−0.11 (−0.95 to 0.59)	

A and/or T, Adenoidectomy and/or Tonsillectomy; WC/H, waist circumference/height ratio; BMI, body mass index.

Results expressed in Z-score, according to the World Health Organization for classification of nutritional status.

a X^2^-test.

b Fisher’s Exact test.

c Mann–Whitney test.

d Student’s *t* test.

When assessing the symptoms of OSA and correlated alterations, such as excessive daytime sleepiness and habitual snoring, it was observed that, of the total sample, 19 (38.78%) had scores ≥7 on the RSD subscale, indicating a high risk for OSA, with an average of 6.35 (SD ± 3.36), with the score ranging from 3 to 15. Snoring was the most common symptom (48.98%, with snoring ≥3×/week) followed by respiratory pauses, which were reported by 18.75% of children and parents (≥1 to 2×/week). The EDS subscale showed that 6 children (12.24%) had positive scores for excessive daytime sleepiness (EDS ≥ 19) with a mean of 8.58 (SD ± 4.88), ranging from 5 to 22. The estimated prevalence of OSA, based on symptoms, was 38.78% with a 95% confidence interval (95% CI) between 25% and 52%; for habitual snoring, it was 48.98% (95% CI) between 34.98% and 62.98% and for EDS it was 12.24% (95% CI) between 3.07% and 21.42%. Table 2 shows the results related to the prevalence of OSA, EDS, nasal obstruction (NO) and habitual snoring (HS).

**Table 2:** Prevalence of Obstructive Sleep Apnea (OSA) symptoms, habitual snoring, Excessive Daytime Sleepiness (EDS) and Nasal Obstruction (NO) in the sample of children with non-syndromic Pierre Robin sequence, according to the score obtained after applying the Sleep Disturbance Scale for Children (SDSC).

Variables	Total sample	95% CI
SDSC (RSD subscale)		
Mean ± standard deviation	6.35 ± 3.36	
Median (25%–75% quartiles)	5.5 (4–7.5)	
Minimum–maximum values	3–15	
SDSC ≥ 7, n (%)	17 (38.78)	(25.13–52.42)
Habitual snoring		
Mean ± standard deviation	3 ± 1.57	
Median (25%–75% quartiles)	2.5 (2–5)	
Minimum–maximum values	1–5	
Snoring ≥3 times a week, n (%)	24 (48.98)	(34.98–62.98)
SDSC (EDS subscale)		
Mean ± standard deviation	8.58 ± 4.88	
Median (25%–75% quartiles)	7 (5–9.5)	
Minimum–maximum values	5–22	
SDSC ≥ 19, n (%)	6 (12.24%)	(3.07–21.42)
CQ-5		
Mean ± standard deviation	3.71 ± 4.72	
Median (25%–75% quartiles)	2 (0–5)	
Minimum–maximum values	0–19	
Positive ≥ 6, n (%)^a^	8 (16.33)	(5.98–26.68)

CQ-5 ≥ 6, high risk of nasal obstruction; SDSC (RSD) ≥ 7, high risk for OSA; SDSC (EDS) ≥ 19, high risk for excessive daytime sleepiness; CI, 95% confidence interval.

Regarding NO, the mean score was 3.70 (SD ± 4.72), ranging from 0 to 19 in the CQ-5 questionnaire. Of the total number of assessed children, 8 (16.33%) had a score ≥6 (Table 2). The most prevalent symptom was oral breathing (20.83%) followed by “stuffy nose” (18.75%), with the prevalence of NO equal to 16.33%, with a 95% confidence interval ranging from 5.98% to 26.68%. As for the assessment of the variables related to the population’s characteristics, age (age range 10–12 years) showed a statistically significant relationship (*p* = 0.009) with the prevalence of NO, and the other variables did not show a significant relationship (*p* > 0.05) with age (Table 1).

Regarding the evaluation of enuresis and symptoms of lower urinary tract dysfunction, it was observed that 8 children (16.33%) of the total sample had enuresis, with a 95% confidence interval of 5.98%–26.68%. It was also identified that for symptoms of lower urinary tract dysfunction, the most frequent complaint was voiding delay (27.08%) followed by voiding urgency (17.78%). Enuresis was the fourth most common symptom in the total sample (Table 3).

**Table 3:** Prevalence of enuresis and symptoms of lower urinary tract dysfunction in the sample of children with non-syndromic Pierre Robin sequence, estimated by the score obtained in the voiding questionnaire (modified VDSS).

Variables	Total sample	95% CI
Enuresis^a^, n (%)	8 (16.33)	(5,98–26,68)
Nocturia^b^	6 (12.5)	
Daytime urinary incontinence, n (%)	5 (10.41)	
Small number of voids (<3×/day), n (%)	9 (18.75)	
Urination delay, n (%)	13 (27.08)	
Urinary urgency, n (%)	11 (22.92)	
Effort to urinate, n (%)	3 (6.25)	
Dysuria, n (%)	2 (4.16)	
Does not evacuate daily, n (%)	13 (27.09)	
Effort to evacuate, n (%)	18 (37.50)	

a Urinary incontinence during sleep, with one or more episodes per month, persisting for the last 3 months.

b Waking up to urinate during sleep (≥1×) more than twice a week for the past 6 months.

Regarding the evaluation between the groups with and without enuresis, there was a statistically significant relationship between family history of enuresis (*p* = 0.006), younger age in years (6–7 years) (*p* = 0.035) and positive score on the VDSS scale (*p* = 0.012), with these factors being associated with a higher prevalence of enuresis. Other clinical variables and characteristics of the sample did not show a statistically significant relationship with a higher prevalence of enuresis or symptoms of lower urinary tract dysfunction (*p* > 0.05).

When analyzing the relationship between the studied variables, a significant relationship was observed between nasal obstruction and OSA symptoms (*p* = 0.001). There was no significant relationship between OSA symptoms and enuresis (*p* = 0.236) and between nasal obstruction and enuresis (*p* = 0.175). The results regarding the comparison of prevalence between studies with a population of children with NSCLP and non-syndromic PRS can be seen in Table 4.

**Table 4:** Comparison of the prevalence of symptoms of obstructive sleep apnea (OSA), habitual snoring (HS), excessive daytime sleepiness (EDS), nasal obstruction (NO) and enuresis obtained in the sample of children with Non-Syndromic Pierre Robin sequence with those obtained in literature studies carried out in a population with non-syndromic CLP and PRS.

Variables	Previous studies			Current study				
	Authors	n (total)	%	n (total)	%	PR	95% CI	*p*
OSA	Moraleda-Cibrian et al., 2014^a^	37	43	48	38.78	0.92	0.55–1.52	0.086
	Silvestre et al., 2014^b^	489	14.7	48	38.78	2.69	1.79–4.05	<0.001
	Fernandes et al., 2018^c^	174	34	48	38.78	0.49	0.34–0.70	0.703
HS	Moraleda-Cibrian et al., 2014	37	44	48	48.98	1.11	0.69–1.78	0.889
	Fernandes et al., 2018	174	62	48	48.98	1.57	1.09–2.28	0.037
EDS	Moraleda-Cibrian et al., 2014	37	48	48	12.5	0.50	0.24–1.02	0.013
NO	Fernandes et al., 2018	174	26	48	16.33	1.00	0.49–2.04	0.871
Enuresis	Fernandes, 2018	174	16.67	48	16.33	1.00	0.49–2.04	0.162

PR, prevalence ratio.

a Study carried out with children with PRS with the PSQ (Pediatric Sleep Questionary) index ≥ 0.33 as the criterion for OSA.

b Study carried out with children with NSCLP having the PSQ (Pediatric Sleep Questionary) as criteria for OSA ≥ 0.33.

c Study carried out with children with NSCLP having SDSC index ≥7 as the criterion for OSA.

## Discussion

The present study aimed to evaluate the relationship between symptoms of obstructive sleep apnea, nasal obstruction and nocturnal enuresis, determining the prevalence of these conditions in schoolchildren with non-syndromic PRS. When assessing the anthropometric and clinical characteristics of the studied population, the predominance of children classified as having normal weight, with no central obesity, and born at term was observed, with the majority showing no associated comorbidities. The comparisons between the groups, evaluating aspects such as WC/H ratio, BMI Z-score, gender, previous adenoidectomy and/or tonsillectomy and presence of secondary palatoplasty showed no statistically significant difference (*p* < 0.05) in the subgroups with and without OSA symptoms, with and without nasal obstruction and with and without enuresis symptoms. This finding shows the lack of interference by these factors on the results.

Among the main characteristics associated with a higher prevalence of OSA in children are obesity, with increased waist circumference, prematurity at birth, enlarged tonsils and/or adenoids and male gender.^33^, ^34^, ^35^, ^36^ In the present study, these variables did not show a significant relationship with the prevalence of OSA symptoms (*p* > 0.05).

The evaluation of the surgical history showed that the 46 children underwent primary reconstructive surgeries of the palate and, 12 had secondary surgeries. The literature shows that palatoplasty may be a risk factor for OSA.^6^, ^7^, ^36^ However, the present study did not show any statistically significant relationship (*p* > 0.05) regarding the performance of secondary palatoplasty, adenoidectomy and/or tonsillectomy between groups with and without apnea symptoms, with and without nasal obstruction and with and without enuresis symptoms.

Using questionnaires, the present study assessed the prevalence of OSA symptoms in the population of children with non-syndromic PRS. A high prevalence of OSA symptoms was identified, with a positive association with nasal obstruction (*p* < 0.05), although most cases of apnea did not show nasal obstruction (63.75%), indicating that another factor may be associated with its causality in this population. The findings indicated the presence of OSA symptoms in 38.78% of the analyzed children, with the main symptoms being snoring, followed by reports of respiratory pauses and breathing difficulties, including noisy breathing and use of accessory muscles.

A high prevalence of RSD has been observed in the population with PRS, being quite higher than that observed in the overall pediatric population, which varies between 1% and 5%.^7^ A study by Moraleda-Cibrián et al., using questionnaires, demonstrated a prevalence of 43%–48% of RSD in children with PRS in the age group of 2–18 years.^21^ More recently, Van Lieshout et al. found, using polysomnographic evaluation, a prevalence of 22% of OSA in the population with syndromic PRS, in the age group from 1 to 18 years old, comparatively higher than in the studied population with non-syndromic cleft lip and palate, in which the prevalence ranged from 14.7% to 18%.^1^, ^17^, ^21^ In turn, a recent study conducted in 2018 identified 34% prevalence of OSA symptoms in a population between 6 and 12 years old with NSCLP, through these questionnaires.^11^ Therefore, it can be observed that children with PRS have a higher prevalence of OSA symptoms than the overall pediatric population and with non-syndromic cleft lip and/or palate.

The high prevalence of RSD and even the occurrence of severe cases of OSA in patients with PRS seems to be related to the reduced mandibular dimensions and associated glossoptosis, conditions that contribute to the narrowing of the UA.^37^ Additionally, the presence of a cleft palate, itself, alters the palatine musculature, impairing the permeability of the UA.^6^, ^38^ Moreover, the effects of repair surgeries, which, on the one hand, promote great improvement in speech and swallowing, have the potential to promote, on the other hand, greater anatomical narrowing of the airway and possible muscle dysfunctions caused by the healing process.^6^, ^7^, ^38^

Additionally, occurrence of habitual snoring (snoring ≥ 3 times a week) was assessed in the analyzed population of children, and a prevalence of 48.98% was observed, as well as excessive daytime sleepiness (SDSC, EDS subscale ≥19), with a prevalence of 12.5%. These variables were analyzed by their previously recognized association with OSA. The observed high prevalence of habitual snoring in a patient with non-syndromic PRS corroborates the data from the study by Moraleda-Cibrian et al. (44%), indicating high rates of habitual snoring in this population, which constitutes an independent risk factor for OSA.^21^, ^36^ The EDS assessment (12.5%) showed a lower prevalence than that reported by Moraleda-Cibrian et al. (48%).^21^ Although reports of excessive daytime sleepiness associated with RSD are common in adult populations, this finding is less prevalent in pediatric populations, even though children with craniofacial anomalies are more prone to EDS.^21^, ^39^

The nasal obstruction symptoms were assessed using the CQ-5 questionnaire. The estimated prevalence of nasal obstruction in the evaluated population, which had only isolated cleft palate, was 16.33%. These values ​​were lower than that found in a study carried out in 2018 (26%)^11^ when assessing children with non-syndromic cleft lip and palate. This finding is justified by the lower involvement of nasal structures in patients with isolated cleft palate, differently from what is observed in clefts that affect the lip and palate together.^11^, ^16^ The most prevalent symptom was oral breathing (20.83%) followed by the sensation of “stuffy nose” (18.75%).

Regarding the evaluation of the variables related to the population’s characteristics, age (age range 10–12 years) showed a statistically significant relationship (*p* = 0.009) with a higher prevalence of nasal obstruction, with the other variables showing no significant relationship (*p* > 0.05). This finding may be associated with the population’s characteristics, such as alterations resulting from facial growth itself, with consequent nasosinusal structural changes, requiring additional studies to confirm and elucidate the observed association.

The relationship between nasal obstruction and OSA is commonly reported in the literature. Nasal obstruction significantly interferes with airflow during sleep and impairs the normal physiological functions of the nasal cavity^10^ being a possible primary factor that leads to pharyngeal collapse, according to the Starling resistor model.^40^ Several studies have indicated a relationship between nasal obstruction and a higher risk of RSD.^10^, ^12^ In this regard, signs of nasal obstruction have been reported in up to 50% of patients diagnosed with OSA,^9^ with nasal obstruction being associated to a higher occurrence of OSA,^13^, ^16^ although a recent study conducted in 2020^14^ did not identify a relationship between nasal dimensions (area and volume) and OSA severity.

The evaluation of enuresis and voiding dysfunction symptoms showed an estimated prevalence of 16.33% in the total sample, being characterized as frequent in 50% of these cases, with a predominance of primary (71.43%) and polysymptomatic (55.55%) enuresis. Regarding the comparison between the groups with and without enuresis, there was a statistically significant relationship between family history of enuresis (*p* = 0.006), younger age (0.035) and positive score on the VDSS scale (*p* = 0.012), as factors associated with greater prevalence of enuresis.^20^ Other clinical variables and sample characteristics did not show a statistically significant relationship with a higher prevalence of enuresis or lower urinary tract dysfunction symptoms (*p* > 0.05).

These results are in agreement with the prevalence rates observed in the overall pediatric population,^41^ which varies from 1.4% to 28% in children aged 6–12 years. It is noteworthy that there was no statistically significant difference between the prevalence of enuresis observed in children with NSCLP and in the sample analyzed in the present study (prevalence ratio — PR = 1.00; *p* = 0.162).^11^ Monosymptomatic enuresis was defined as not associated with lower urinary tract dysfunction symptoms and the polysymptomatic type was defined as associated with lower urinary tract dysfunction symptoms, according to the score obtained at the VDSS.^11^

Currently, a still poorly elucidated relationship has been observed between obstructive sleep apnea and enuresis,^42^, ^43^ so that the correction of the causes involved in OSA and its remission tend to improve the severity and frequency of enuresis, although the physiological mechanism involved in this correlation is not yet fully elucidated.^44^, ^45^ In the present study, however, no significant correlation was observed between OSA symptoms and enuresis, and PRS as a greater risk for enuresis. On the other hand, there was a significant correlation between nasal obstruction and symptoms of obstructive sleep apnea, in agreement with what was observed in a study carried out in 2018^11^ in a population of children with non-syndromic cleft lip and palate.

The starting point of the present study was to compare the patient population data with previously studied NSCLP,^11^ in patients with non-syndromic PRS regarding the prevalence of OSA symptoms, nasal obstruction and enuresis. These, due to mandibular retraction and glossoptosis, are at increased risk for OSA,^21^ and it was hypothesized that the occurrence of symptoms associated with OSA would be higher in the present study. However, there were no statistically significant differences regarding the prevalence of OSA symptoms (PR = 0.49; *p* = 0.703), nasal obstruction (PR = 1.00; *p* = 0.871) and enuresis (PR = 1.00; *p* = 0.162) with the results obtained in the previous study with children with NSCLP.^11^

Regarding the habitual snoring, when comparing to the results obtained in 2018 in children with NSCLP,^11^ there was a statistically significant difference (*p* < 0.05), with 48.98% observed in the current study *versus* 62%, and although a lower percentage was observed in the present study, it should be noted that the study with which the comparison was made had a 3.5-fold larger sample.^11^

The strengths of the present study include the number of participants, when compared to other studies that evaluated children with non-syndromic PRS, the adoption of strict inclusion criteria and the sample homogeneity, allowing the ruling out of interference of anthropometric and surgical characteristics on the findings.

The study limitations were the use of subjective analysis instruments (questionnaires), which even though are frequently use in the literature having adequate test and retest reliability, do not constitute the gold standard for the evaluation of OSA and NO.^11^ Studies that objectively assess the occurrence of OSA and nasal obstruction in patients with non-syndromic PRS, through polysomnography, acoustic rhinometry and/or rhinomanometry, should be developed in the future, aiming to elucidate this complex issue.

## Conclusion

The present study showed a high prevalence of symptoms of obstructive sleep apnea and habitual snoring in the population of children of school age with non-syndromic Pierre Robin sequence. A correlation was observed between the presence of nasal obstruction and obstructive sleep apnea symptoms. Prevalence rates of excessive daytime sleepiness, nasal obstruction and enuresis did not differ from those found in the overall pediatric population. A family history of enuresis, younger age and a positive score on the “voiding dysfunction score system scale” were associated with a higher prevalence of enuresis. Contrary to the initial hypothesis, the presence of non-syndromic Pierre Robin sequence, symptoms of obstructive sleep apnea and nasal obstruction were not risk factors for the occurrence of enuresis in the studied population.

## Conflicts of interest

The authors declare no conflicts of interest.
